# Multifaceted Transcriptional Network of Estrogen-Related Receptor Alpha in Health and Disease

**DOI:** 10.3390/ijms24054265

**Published:** 2023-02-21

**Authors:** Catherine Cerutti, Jing-Ru Shi, Jean-Marc Vanacker

**Affiliations:** Institut de Génomique Fonctionnelle de Lyon, Université de Lyon, CNRS UMR5242, Ecole Normale Supérieure de Lyon, 69342 Lyon, France

**Keywords:** nuclear receptor, transcriptional regulator, target gene, regulatory network, metabolism, cancer

## Abstract

Estrogen-related receptors (ERRα, β and γ in mammals) are orphan members of the nuclear receptor superfamily acting as transcription factors. ERRs are expressed in several cell types and they display various functions in normal and pathological contexts. Amongst others, they are notably involved in bone homeostasis, energy metabolism and cancer progression. In contrast to other nuclear receptors, the activities of the ERRs are apparently not controlled by a natural ligand but they rely on other means such as the availability of transcriptional co-regulators. Here we focus on ERRα and review the variety of co-regulators that have been identified by various means for this receptor and their reported target genes. ERRα cooperates with distinct co-regulators to control the expression of distinct sets of target genes. This exemplifies the combinatorial specificity of transcriptional regulation that induces discrete cellular phenotypes depending on the selected coregulator. We finally propose an integrated view of the ERRα transcriptional network.

## 1. Introduction

In eukaryotes, regulation of gene expression relies on a combinatorial interplay between DNA-binding transcription factors (TFs) and non-DNA binding coactivators or corepressors. Among non-DNA-binding co-regulators, those involved in histone modifications are of importance to control chromatin accessibility and the dynamics of the transcriptional process [1]. The coordinated activity of all these cooperating components results in specific spatiotemporal effects on target gene expression [2,3,4,5]. Pairwise interactions between TFs or between TF and non-DNA binding coactivators can be demonstrated at the protein level [6,7,8]. However, simultaneous cooperative recruitment of more than two transcriptional partners may occur and is currently difficult to demonstrate experimentally.

Nuclear receptors (NRs) form a family of transcription factors whose activities are generally controlled by the recruitment of specific, endogenous ligands. NRs are present in all animals and 21 of them have been identified in *D. melanogaster* vs 48 in *H. sapiens*. NR proteins are organized in a similar manner. They comprise an N-terminal domain that can contribute to ligand-independent transcriptional activities, a centrally located DNA-binding domain (DBD) containing two Zn fingers, a hinge domain and a C-terminally located Ligand Binding Domain (LBD). The LBD is also involved in receptor homo- or heterodimerization. Furthermore, ligand recruitment induces a conformational change in the LBD that allows interactions with transcriptional cofactors, leading to the modulation of target gene expression. The DBD and, to a lesser extent, the LBD are the most conserved domains of NRs across evolution. The transcriptional activities exerted by NRs also require a large set of proteins to modulate chromatin structure and to recruit the basal transcription machinery. As for most of the TFs, the involvement of cofactors is both dynamic and hierarchical. Primary cofactors have been proposed as those directly binding to NRs to enhance their functions. Secondary cofactors could be those recruited to the promoter through contact with a primary coactivator or corepressor, thus enhancing or inhibiting NR functions, respectively [9].

The Estrogen-Related Receptors (ERRα, β and γ in mammals) form a subfamily of orphan (i.e., lacking an identified natural ligand) NRs. They are expressed in several tissues during embryologic development and in the adult, and display various physiological and pathological functions [10,11,12,13]. The ligand-independent transcriptional activity of ERRs has been noted for several years. This is apparently due to the presence of particular amino acid side chains in the putative ligand binding pocket that lock the LBD in an active conformation and allows constitutive contacts with co-regulators [14]. Due to the lack of natural ligands that directly regulate the activities of ERRs, it is thought that their transcriptional activity is mediated by the recruitment of coactivators and corepressors [14,15,16]. The necessary presence of these cofactors makes their participation in diverse ERR-centered networks instrumental in the cellular effects of ERRs. It should however be mentioned that synthetic compounds have been identified that promote or restrict the transcriptional activities of ERRs. Some crystallographic studies have suggested that these compounds may alter the conformation of the receptors, compromising the recruitment of co-regulators.

The most-studied member of the ERR family, ERRα, is involved in various functions related to energy metabolism in tissues displaying high energy demands, such as liver, muscles, adipose tissues or heart [11,17,18]. It has also important roles in osteogenesis, immunity, brain functions and tumorigenesis [19,20,21]. In cancer, ERRα has been shown to control proliferation, metabolism, resistance to hypoxia, angiogenesis and cell migration and invasion [12,22,23,24,25]. Work from various laboratories suggests that these specific ERRα activities may be controlled by interaction with dedicated cofactors expressed in given tissues and that thereby control the phenotypic output of ERRα activities. The main purpose of this review is to provide a detailed understanding of the ERRα transcriptional network derived from work over the past decades. Special emphasis is given to the relationship between the ERRα co-regulators and the cellular functions that they specifically modulate through the receptor.

## 2. Transcriptional Activities of ERRs

Many efforts have been devoted these last 25 years to dissect the mechanisms underlying the transcriptional activities of NRs. Co-regulatory proteins have been identified, and in general they are components of multi-protein complexes that contain associated chromatin remodeling and/or histone modifying proteins [26]. Co-regulators generally possess LXXLL motifs (where L is leucine and X is any amino-acid residue), also known as nuclear receptor boxes, which enable their interaction with NRs. For most of them, these interactions have been shown to be ligand dependent. For orphan NRs such as HNF-4, Nur77, RORs, TLX or ERRs, a number of papers each focusing on one member of this subfamily have been published suggesting several interacting cofactors, including those that interact with ligand-dependent NRs [26].

Most NRs bind DNA as dimers, either as hetero- or as homodimers, on DNA sequences organized as two half-sites (with AGGTCA as a consensus sequence) with specific orientation and spacing. For instance, the thyroid hormone receptor-RXR heterodimer mostly binds to the so-called DR4 (Direct Repeat 4), with AGGTCAnnnnAGGTCA as a consensus sequence (n is any nucleotide). On another hand, the Estrogen Receptor α (ERα) homodimer binds to AGGTCAnnnTGACCT, an inverse repeat of half sites separated by three nucleotides. In contrast, ERR-response elements (ERREs) are composed of a single half-site generally preceded by a CA-containing sequence. Yet, the sequence-dependent DNA shape of the binding site influences the recruitment of homodimers of ERRα through their DBD. This is achieved by the promotion of the right conformation of the two subunits on DNA for cooperative interaction and dimer stabilization [27]. Furthermore, interaction with a co-activator may induce an allosteric change in ERRα that allows stable dimerization and DNA binding of both receptor DBDs [28].

**Figure 1:**
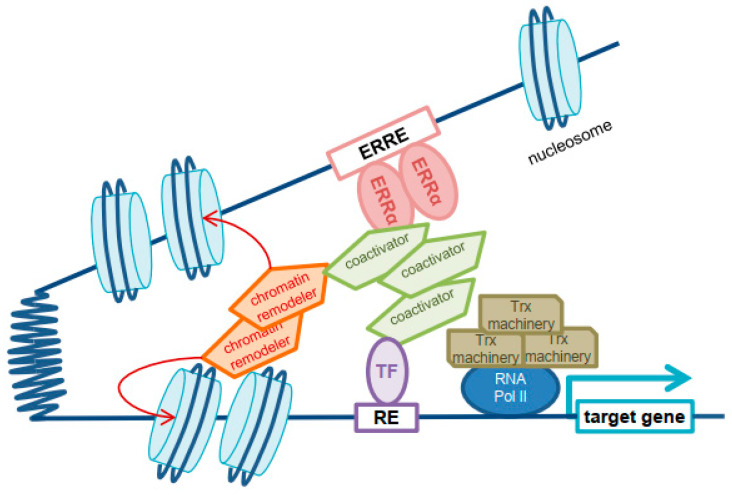
Schematic model of transcriptional regulation operated by ERRα and its coactivators. The estrogen-related response element (ERRE) is often located at a distance from the target gene promoter. Chromatin remodelers allow access of ERRα to nucleosomal DNA. TF: transcription factor; RE: response element; Trx: transcription.

ERREs are often located at a distance from the Transcriptional Start Site (TSS) of their target genes (about 75% at >1 kb upstream) in introns or in distal intergenic regions where coactivators can be directly recruited by ERRα [29]. Recent literature has suggested that a cooperating TF recruited to the TSS could be important to bridge the ERRE-bound ERRα-cofactor complex to the TSS where their molecular effect is exerted [30] (Figure 1; see below).

## 3. General Co-Regulators of ERRs

The p160 coactivator family members (SRC-1/NCoA1, SRC-2/GRIP1/NCoA2, and SRC-3/pCIP/AIB1/ACTR/NCoA3) have been early associated with NRs and the understanding of their importance has grown over time [31]. The three members of this family interact with the activation region of the LBD of NRs and ERRs have been shown to be co-activated by all p160 proteins [32,33]. For instance, in breast carcinoma, NCoA3 has been suggested as a major coactivator of ERRα with binding to the ERREs of ERRα target genes and transcriptional regulation of transfected ERRα-responsive promoters [34].

The p160 coactivators can bind to two other types of coactivators, CREB-binding protein (CBP) and p300, two closely homologous proteins known as p300/CBP family, as well as to p/CAF (p300/CBP-associated factor) [35,36,37]. These proteins act as co-regulators for a wide variety of TFs and also as major lysine acetyltransferases [38,39]. Their various biological functions and how disruption of these functions by mutations and alterations in expression or subcellular localization contributes to cancer phenotype has been more recently reviewed [40]. Although no interaction between ERRs and p300/CBP has been reported, an interaction between ERRα and p/CAF has been revealed in vitro and in mouse liver [41]. This results in the regulation of ERRα transcriptional capacities through unusual mechanisms. Indeed, p/CAF acetylates four Lys residues in the DBD of ERRα. As a consequence, the DNA binding capacity and transcriptional activities of ERRα are strongly reduced. In contrast, SIRT1 and HDAC8 deacetylate the p/CAF-acetylated Lys residues of ERRα resulting in increased DNA binding. The authors also suggest that ERRα acetylation is likely to act in combination with other post-translational modifications, such as phosphorylation or sumoylation, to fine-tune the receptor’s activities. Altogether, this provides an efficient on and off mechanism to regulate the activities of ERRα.

Proline, glutamic acid, and leucine-rich protein 1 (PELP1) is a scaffolding protein with several motifs commonly found in co-regulators that has been ascribed to a large number of cellular functions, including regulation of NR signaling and cross-talk [42]. PELP1 has been shown to interact with ERRα and proline-rich nuclear receptor co-regulatory protein 2 (PNRC2) in the transcriptional activation of aromatase in breast cancer cells [43]. Interestingly, PNRC2 has been reported to modulate the transcriptional activation of other NRs, in particular SF1 and ERRγ, suggesting a general feature exerted by this factor [44,45].

Several corepressors have also been described for ligand-regulated, as well as orphan, NRs. Nuclear receptor corepressor (NCoR1) and the highly similar silencing mediator of retinoic and thyroid receptor (NCoR2/SMRT) were the first identified ones, based on their ability to mediate transcriptional repression of thyroid hormone receptor and RARs. These cofactors mediate transcriptional repression by bridging histone deacetylases (HDACs), in particular HDAC3, to NRs in the absence of their corresponding ligands [46,47]. In breast cancer cells, NCoR1 represses a number of negative ERRα –LSD1 targets, but does not act on other (i.e., non-LSD1 dependent) ERRα targets, suggesting a contribution to the regulation of a specific subset of targets [48]. NCoR1 has been identified as a key physiological regulator of muscle mass and function, through its association with MEF2, PPARβ/δ, and ERRα [49].

The receptor-interacting protein 140 (RIP140), encoded by *NRIP1* gene, is one of the first proteins that have been identified as recruited by hormone-bound NRs. Strikingly, this protein mainly acts as a direct corepressor of NRs [50] but may alternatively regulate their activity by competing the recruitment of coactivators such as SRC-1 as demonstrated in mammalian cells [51]. RIP140′s activity as a corepressor of ERRs was established as depending on the regulatory elements present in the target promoters [52]. However, this study also suggested that RIP140 can increase the activation exerted by ERRα and ERRγ on SP1-binding sites through a mechanism that involves histone deacetylases. Although this remains to be documented in vivo, this suggests that RIP140 can act on ERRs as a coactivator or as a corepressor, depending on the DNA context.

**Figure 2:**
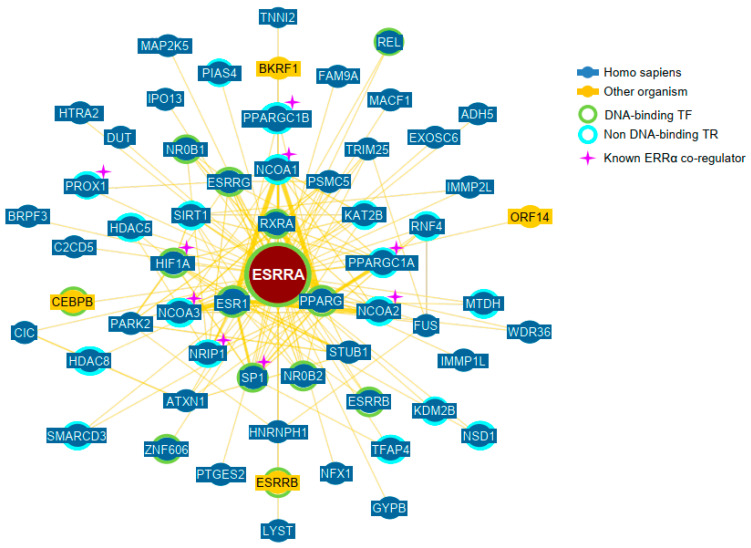
Network of ERRα interacting proteins. The network was obtained from the BioGRID database (https://thebiogrid.org/) on 17 December 2021. The yellow lines joining proteins indicate association with physical evidence. TF: transcription factor; TR: transcriptional regulator.

Fifty-eight proteins interacting with ERRα are reported in the BioGRID database (https://thebiogrid.org) that collects protein–protein interactions from a number of experimental studies. The BioGRID-built network includes transcriptional regulators as well as enzymes, structural or RNA-binding proteins (Figure 2). Among the 30 transcriptional regulators, 9 are part of the pre-cited co-regulators. PNRC2 and NCoR1/2 do not appear in the network suggesting that their co-regulatory role is indirect and occurs through another factor.

## 4. Transcriptional Activity of ERRα in Healthy Conditions

### 4.1. Bone Development

Effects on bone status have been shown for ERRα and ERRγ. Using complete knock-out (KO) mouse models, ERRα was shown as an activator of bone loss during ageing [53,54]. This observation was extended to bone loss resulting from ovariectomy that is also induced by ERRα. This bone loss is indeed abolished in female mice in which ERRα is specifically knocked out in osteoblasts (bone forming cells) [20]. Consistently, it appears that ERRα is an inhibitor of osteoblast differentiation, as observed in vivo and in cell cultures [53,54]. However, the effects of ERRα on osteoblast differentiation are complex and could depend on the presence of the co-regulators PGC-1α and PGC-1β [21]. How ERRα acts in the absence of PGC-1 proteins (as is the case in the early stages of osteoblast differentiation) and through which co-regulators is currently undocumented. Similarly, ERRγ has been identified as anti-osteogenic in bone and pro-osteogenic in the vasculature and no co-regulator was identified that modulates these activities [55,56].

### 4.2. Brain Functions

The transcriptional activity of ERRα was also highlighted in the brain with an important regulatory role in response to social challenge in mice [57]. In addition, cross-talk between ERs and ERRs is documented in the brain, as well as a potential beneficial role of ERRα in Alzheimer disease [58,59]. However, no ERRα coactivator has yet been proposed that could document the mechanism of the receptor’s effect in this context.

### 4.3. Interactions of ERRα with the Immune System

Several studies have shown that ERRα promotes innate host defense. The receptor was first identified as a transcriptional regulator of effector T lymphocytes metabolism [60] and as a transcriptional and post-translational activator of autophagy-related genes via a feed-forward loop with the deacetylase SIRT1 [61]. In this context, ERRα was identified as a target of the NR Nur77 (encoded by the *NR4A1* gene) that represses several TFs known to regulate T cell metabolism following activation [62]. ERRα also represses Toll-like receptor (TLR)-induced inflammation [63], mostly via fine-tuning of metabolic reprogramming in macrophages. Again, no data about any transcriptional coactivator of ERRα in this field have currently been published.

### 4.4. Cellular Metabolism: Role of PGC-1α and β

The first coactivator of ERRα that has been identified is PGC-1α (PPARgamma Co-activator 1α) in the frame of its involvement in mitochondrial energy metabolism [64,65]. PGC-1α has been extensively studied in humans, in health and disease situations. It has been characterized as a master regulator of cellular energy metabolism, including adaptive thermogenesis mediated by multiple transcription factors, such as the NR PPARγ [66]. Work by different laboratories has next shown that ERRα is also instrumental in the activities of PGC-1α in tissues with high energy demand, such as skeletal muscle, heart, liver, or brown adipose tissue [64,67,68]. For instance, ERRα is important for the PGC-1α driven regulation of energy metabolism in cardiac and skeletal muscle [69] and is required for the induction of Ucp1 expression by PGC-1α in the brown adipose tissue [70]. Furthermore, mice lacking ERRα are impaired for thermogenic adaption [71,72]. In addition, PGC-1α also positively regulates ERRα expression thus forming a feed-forward loop [65,73].

PGC-1β has been shown to interact with ERRα and NRF1 to induce several key genes of mitochondrial biogenesis and respiration during differentiation of C2C12 mouse myoblast cells in skeletal myotubes [74]. In mouse heart, ERRα/γ-responsive promoters of metabolic target genes are also enriched for NRF1 as well as for CREB or STAT3 binding sites [75]. Both coactivators PGC-1α and PGC-1β can also mediate the transcriptional activities of ERRα and ERRγ in cancer [76,77]. As such, involvement of the receptors and PGC-1α or β has been documented in the metabolic shift from oxidative to aerobic glycolysis, known as the Warburg effect [78,79].

Several factors have been shown to modulate the transcriptional activity of the ERRα/PGC-1 complex, such as NCoR1, an important modulator of energy metabolism in several tissues. In skeletal muscle, competition between NCoR1 and PGC-1α in the antagonistic regulation of ERRα activity has been proposed for adaptation of oxidative metabolism to physical activity or caloric restriction [80].

The prospero homeobox PROX1 is a TF previously known to regulate the activity of several nuclear receptors, mostly NR5 family members or HNF4A in the liver [81]. PROX1 has been identified as a negative modulator of ERRα/PGC-1α energetic functions in mouse liver [82]. As a TF, PROX1 shares targets with ERRα and interacts directly with PGC-1α [83]. This study also showed a cross-talk between ERRα, PROX1, and BMAL1 (an instrumental factor in the establishment of the circadian cycle) in the rhythmic control of metabolic genes. It should also be reminded that the expression of ERRα is itself under the control of the circadian clock [84]. Together with the association with BMAL, this may provide a reinforcement of the circadian control over gene expression.

A direct interaction of the SP1 TF with the three ERRs has been identified in vitro and in human cancer cells. ERRs are able to activate transcription of some targets through SP1 binding sites as pointed above [54]. In addition, the recruitment of the SP1 protein adjacent to ERRα-binding element was identified in muscle cells as one of the conditions preventing the interaction between PGC-1α and ERRα [85].

## 5. Transcriptional Activity of ERRα in Cancer Progression and Cell Migration

In cancers from different tissues (breast, ovary, prostate, colon, etc.), a strong ERRα expression has been correlated with a poor prognosis [reviewed in 12,23]. Consistently, important roles of ERRα in the promotion of cancer progression have been documented in the past decades, suggesting that inactivation of the receptor may be beneficial against cancers. For some of these functions, transcriptional co-regulators have been identified.

ERRα is involved in the regulation of the proliferation of cell lines derived from breast cancer (such as MCF7 or MDA-MB231) or prostate cancer (LNCaP or PC3) as well as of mammary cancer cells xenografted on Nude mice [86]. Co-regulators of ERRα in this cellular effect have not been investigated.

The receptor is also involved in the adaptation of prostate cancer cell lines (LNCaP or PC3) to hypoxia and in the induction of angiogenesis [87]. HIF-1α plays a major role in the regulation of cancer cell metabolism that is reconfigured towards lactate production following aerobic glycolysis through the Warburg effect [88]. ERRs have been shown as essential cofactors of HIF-1α mediating the response to hypoxia [89]. In addition, ERRα physically interacts with HIF-1α in prostate cancer cells [86]. Together, these two factors activate the expression of genes regulating metabolism (such as LDHA or PKM2) and blood vessel growth (such as VEGF or EPO). As developed above, ERRs cooperate with PGC-1 factors in the (dys)regulation of cancer cell metabolism. It has been shown that the ERRα-PGC-1α complex regulates the expression of VEGF in cooperation with HIF-1α.

Other activities of ERRα in cancer cells have been documented that do not depend on PGC-1 proteins. This is for instance the case of phenomena contributing to cell migration. These activities have first been identified in physiological situations. This is the case of zebrafish embryonic development where ERRα inactivation reduces cell motility [90], as well as of the capacity of activated macrophages to invade the peritoneal cavity in vivo which is reduced in ERRαKO mice [24]. In cell cultures, ERRα regulates the dynamics of actin network and of focal adhesions which anchor cells to their substrate [25]. These phenomena need to be synchronized for cell migration which is promoted by ERRα [22,24]. In addition, the receptor increases the capacity of cells to invade the extracellular matrix [30,48]. Recent work has shown that ERRα cooperates with different transcriptional coactivators to regulate cell migration and invasion.

### 5.1. Role of LSD1 as an ERRα Coactivator

Our team has identified the histone lysine specific demethylase 1 (LSD1) as an ERRα coactivator that promotes the migration of breast cancer cells [48]. As is the case for ERRα, high expression of LSD1 has been identified by others as a poor-prognosis marker in breast cancers [91,92]. LSD1 can act as a transcriptional repressor by demethylating H3K4me2 or as a transcriptional activator by demethylating H3K9me2 [93,94,95]. The conditions governing the balance between these two activities are unclear to date. However, in vitro experiments have shown that ERRα switches the activities of LSD1 from H3K4me2 to H3K9me2 demethylation [48]. In breast cancer cells, this biochemical activity occurs at the TSS of target genes commonly activated by ERRα and LSD1 and which are involved in the promotion of cell migration. However, the ERRα-LSD1 complex is bound to DNA on enhancer-localized ERREs. Additional work has shown that the NRF1 TF recruits the ERRα-LSD1 complex to the TSS of target genes involved in the regulation of cell invasion [30]. This is for instance the case of MMP1 (Matrix Metalloprotease 1), whose TSS displays an increase in H3K9 methylation in the absence of ERRα or LSD1. As a consequence, inactivation of ERRα, LSD1 or NRF1 leads to reduced degradation of the extracellular matrix and decreased cell invasion, a defect that can be rescued by MMP1 re-expression [24,30,48].

### 5.2. Recently Identified ERRα Coactivators

Recent results of the team identified possible transcriptional regulators associated with ERRα using unbiased statistical expression models of ERRα-activated genes across various breast cancer cells [29]. Gene expression modeling is a suitable computational way to propose potential transcriptional regulators, even non-DNA-binding ones, selected from a large set. Indeed, using RNA expression data, those regulators contributing to the expression of some genes in association with a specific TF gene, as ERRα, are potential co-regulators of this TF. Interestingly, this approach can unveil several potential co-regulators in the same experimental context. Among those identified, *DDX21*, *MYBBP1A*, *NFKB1*, and *SETD7* were validated in breast cancer cells as modulators of some ERRα-activated genes. Moreover, SET7 was further confirmed as a transcriptional partner of ERRα, with which it physically interacts and regulates the expression of target genes involved in cell motility. Consistently, both factors are necessary to induce orientated cell migration.

**Figure 3:**
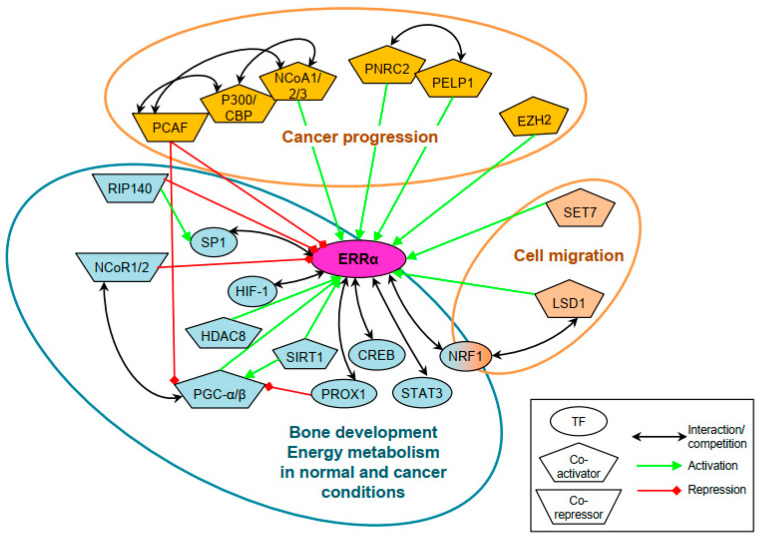
Transcriptional co-regulators of ERRα and related cellular functions. Coactivators and corepressors of ERRα already identified in various conditions through various mechanisms are shown. They are involved in three main cellular functions: bone development, energy metabolism, and cancer development.

It is likely that all transcriptional co-regulators of ERRα have not been characterized to date. However, the identified co-regulators are very diverse as are the cellular functions that are regulated by ERRα in association with a particular cofactor (see Figure 3).

### 5.3. Indirect Modulations of ERRs Transcriptional Activity: Effect on Receptor Expression

The enhancer of zeste homolog 2, EZH2, is a subunit of the Polycomb repressor complex 2 that acts as a histone methyltransferase. Several lines of evidence have implicated EZH2 in the development and progression of a variety of cancers and it has become a potential therapeutic target [96,97]. Functional interaction of EZH2 with ERRs was evidenced in gastric and breast cancers [98,99]. Indeed, EZH2 binds to all ERR promoters and represses their expression. Combined treatment of an EZH2 inhibitor and ERRγ agonist displays a synergistic suppressive effect on gastric cancer progression [98]. In breast cancer, EZH2 was identified as a regulator of ERRγ activities in a methyltransferase-dependent manner [99].

Various post-transcriptional or post-translational modifications exerted by diverse compounds can affect the stability and transcriptional activity of ERRα. Detailed knowledge on this topic is summarized in a recent review on the regulation of the expression of ERRs [100].

Reduction of ERRα transcript abundance induced by various miRNAs (miR-125a, miR-137, miR-135a, miR-497) has been shown during adipocyte differentiation or cancer cell migration [101,102,103,104]. In contrast, stabilization of the ERRα protein by high levels of LSD1 has been shown in breast cancer cell lines [105]. LSD1 protects ERRα from proteasome-dependent degradation, independently of its demethylase activity, and without any effect on ERRα mRNA.

In addition, some synthetic compounds modulate the transcriptional activities of ERRs such as bisphenol A, diethylstilbestrol, and 4-hydroxytamoxifen that act mostly on ERRγ and ERRβ as agonists or antagonists [106]. However, these compounds all display minimal activity on ERRα. Proteasome-dependent degradation of ERRα can be induced by its synthetic inverse agonist XCT790 that also blocks the transcriptional activity of the receptor [107]. This degradation effect is potentiated by the ERα antagonist ICI182,780 (also known as Fulvestrant), that is used in the treatment of certain breast cancers.

## 6. Summary and Perspectives: Specificity of Co-Regulators for ERRα Targets and Cellular Functions

**Figure 4:**
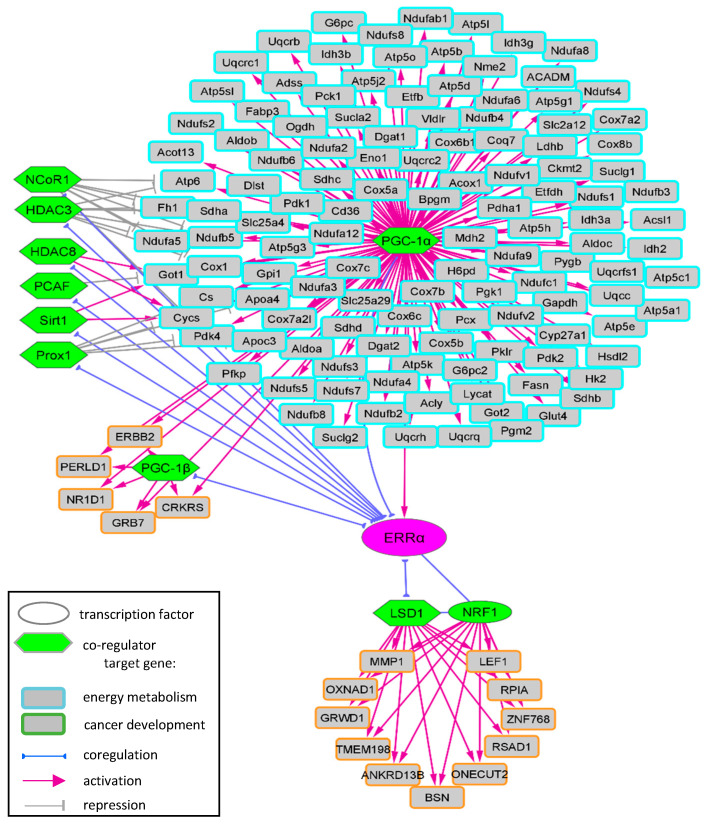
Transcriptional network of ERRα-PGC-1 and ERRα-LSD1 complexes. ERRα coactivators PGC-1 and LSD1, alone or in association with other co-regulators, control the expression of specific genes involved in energy metabolism or cancer development.

The transcriptional activity of ERRα relies on the recruitment of a number of co-regulators that have been identified in various tissues or cells and in health or disease states. In addition, various in vitro and in vivo experiments were used to disclose the interaction between ERRα and its co-regulators as well as their effects on ERRα target genes. The identified target genes mostly differ across studies, possibly reflecting the different tissues/cells that were used to characterize these targets. This suggests that each ERRα-co-regulator complex modulates the expression of specific sets of genes and thus exerts selective phenotypic effects. PGC-1s are the main ERRα coactivators turning the effects of ERRα towards cellular energy metabolism in healthy tissues with high-energy demand or in tumors. The role of ERRα in cancer progression appears mediated by different coactivators to control other specific target genes. Figure 4 recapitulates the target genes identified from PGC-1 and LSD1 studies (see all the co-regulators and their identified target genes in Table 1 and Figure S1).

In summary, orphan nuclear receptors ERRs represent a complex model of transcriptional regulation. In particular for ERRα, the diversity of co-regulators reflects the diversity of target genes as part of a diversity of cellular functions. However, co-regulators have been identified one at a time in various conditions and we are still lacking a global view for a given condition. Notably, our knowledge on the multiple ERRα coactivators and their physical interaction or cooperation needs to be improved. Because multiple-protein interactions cannot yet be simultaneously investigated with current molecular or cellular biology approaches, this challenging issue could be first addressed by computational approaches [108,109]. In addition, the recruitment of numerous transcriptional coactivators occurs through a specific dynamics that remains to be explored for ERRs [110]. Such new data could lead to a better understanding of the different mechanisms underlying the effects of ERRα for designing new context-specific compounds in the treatment of diseases in which ERRα is expressed and involved.

## Figures and Tables

**Table 1:** Summary of known ERRα coregulators and their associated target genes.

Coregulator	ERRα Coregulation Type	Tissue/Cell or Technique	ERRα Target Genes	Methods for ERRα Interaction	Reference
SRC-1/NCoA1, SRC-2/GRIP1	coactivation (NR family)	molecular, in vitro	none	PPI via yeast two-hybrid assay and GST pull-down assays	Hong et al. 1999 [32]
SRC-1/NCoA1	coactivation	in vitro, mouse embryonic stem cells	*TFF1/pS2*; cell cycle: *USP17L2/Dub3*	gene expression by RT-qPCR, transient transfection and reporter assays	van der Laan et al. 2014 [33]
SRC-3/NCoA3/ AIB1	coactivation	human breast cancer, human cells (HEK293)	aromatase *CYP19A1*, *TFF1/pS2*, lactoferrin *LTF*	PPI via mammalian two-hybrid assay, in vivo coIP, ChIP	Heck et al. 2009 [34]
SRC-1/NCoA1, PGC-1α	coactivation	crystallographic analysis structure of the human ERRα LBD	none	cocrystallization	Kallen et al. 2004 [16]
PGC-1α	coactivation	human heart	fatty acid oxidation: *ACADM*	PPI via yeast two-hybrid assay	Huss et al. 2002 [64]
PGC-1α	coactivation	mouse heart	lipid metabolism: *Acsl1*, *Cd36*, *Acox1*; TCA cycle: *Idh2*; oxidative metabolism: *Ndufb3*, *Cox8b*, *Atp5e*, *Ckmt2*; other metabolic processes: *Hsdl2*, *Lycat*, *Adss*, *Nme2*	gene expression in ERRα KO hearts	Huss et al. 2007 [17]
PGC-1α, PGC-1β	coactivation	mouse mammary tumor, human breast cancer cells (SKBr3)	*ERBB2*, *CRKRS*, *PERLD1*, *GRB7*, *NR1D1*		Deblois et al. 2010 [76]
PGC-1α, PGC-1β	coactivation	human cancer	mitochondrial biogenesis and energy metabolism		Deblois et al. 2013 (review) [23]
PGC-1α, PGC-1β	coactivation	various human tissues and conditions	metabolic genes and cellular energy metabolism		Huss et al. 2015 (review) [13]
PGC-1α + PCAF or HDAC8, Sirt1	repression by acetylation (PCAF) and activation by deacetylation (HDAC8 or Sirt1)	mouse liver, mouse hepatocytes, human COS-1 fibroblasts, HEK293	*Got1*, *Cycs*		Wilson et al. 2010 [41]
PGC-1α + NCoR1 via HDAC3	antagonization of PGC-1α-mediated coactivation of ERRα by NCoR1	mouse skeletal muscle, C2C12 myoblasts	oxidative metabolism: *Sdha*, *Ndufa5*, *Ndufb5*, *Fh1*, *Cox1*, *Atp6*	gene expression in muscle specific NCoR1 KO mice	Pérez-Schindler et al. 2012 [80]
PNRC2	coactivation	human mammary gland, human breast cancer cells (SKBr3)	aromatase *CYP19A1*	PPI via yeast two–hybrid and GST pull-down assays, coIP	Zhou et al. 2000 [44]
PELP1 + PNRC2	coactivation	human breast cancer cells (MCF7)	aromatase *CYP19A1*	PPI via yeast two-hybrid assay, in vitro reporter gene assays, ChIP	Rajhans et al. 2008 [43]
SP1	ERRα competitor, affects ERRα-PGC-1α targets	mouse muscle cells (C2C12), muscle-specific PGC-1α KO or transgenic mice	*Pdpr*, *Lrpprc*, *Acot13*, *Mul1*	ChIP-seq, microarray gene expression, DNA-binding motifs by bioinformatics	Salatino et al. 2016 [85]
LSD1	coactivation	human cancer cells (MDA-MB-231, HeLa), human embryonic cells (HEK293T)	cell migration genes: *ANKRD13B*, *BSN*, *GRWD1*, *LEF1*, *MMP1*, *ONECUT2*, *OXNAD1*, *RPIA*, *RSAD1*, *TMEM198*, *ZNF768*	RNA-seq gene expression after siRNA, PPI by GST pull-down assays, coIP, PLA, ChIP	Carnesecchi et al. 2017 [48]
LSD1 + NRF1	coactivation complex	human cancer cells (MDA-MB-231), human embryonic cells (HEK293T)	cell migration genes: *ANKRD13B*, *BSN*, *GRWD1*, *LEF1*, *MMP1*, *ONECUT2*, *OXNAD1*, *RPIA*, *RSAD1*, *TMEM198*, *ZNF768*	ChIP, RT-qPCR gene expression, DNA-binding motifs by bioinformatics	Zhang et al. 2018 [30]
SET7	coactivation	human cancer cells (MDA-MB-231)	*CELF1*, *ESM1*, *FAM155B*, *KLHL18*, *LMNB1*, *NFATC2*, *PHACTR1*, *PHLDB2*, *PPM1E*, *RAI14*, *SAMD12*, *SAMD4A*, *SFTA1P*, *SNCAIP*	RNA-seq and RT-qPCR gene expression after siRNA, coIP, PLA, HA-SET7 cells overexpressing SET7, cell wound healing	Cerutti et al. 2022 [29]
RIP140/NRIP1	corepression via HDAC1, increase of Sp1-mediated transactivation of ERRα	human cancer cells (HeLa, MCF-7)	*pS2/TFF1*, *TRα*, *p21*, *SRY*	PPI via GST pull-down assays and luciferase activity from reporter plasmids	Castet et al. 2006 [52]
EZH2	coregulation (binds ERRα promoter)	human breast cancer samples, human breast cancer cells (MCF-7, T47D, MDA-MB-231)	*ERRα*, *ERRβ*	RT-qPCR gene expression, ChIP	Kumari et al. 2018 [99]
Prox1	negative modulation of ERRα-PGC-1α	mouse liver + human liver cells (HepG2)	metabolic genes: *Pdk4*, *Cs*, *Cycs*, *Apoc3/Apoa4*	PPI via GST pull-down assays and yeast two-hybrid assay, coIP	Charest-Marcotte et al. 2010 [82]
NCOR1	corepression	mouse skeletal muscle, human cells (HEK293)	*Pdk4*	gene expression in skeletal muscle specific NCoR1 KO mice, coIP (negative result)	Yamamoto et al. 2011 [49]

NR: nuclear receptor; PPI: protein-protein interaction; GST: glutathione-S-transferase; coIP: co-immunoprecipitation; ChIP: chromatin immunoprecipitation.

## Data Availability

Not applicable.

## Supplementary Materials

The following supporting information can be downloaded at: https://www.mdpi.com/article/10.3390/ijms24054265/s1.
