# Supplementary material for: Multifaceted Transcriptional Network of Estrogen-Related Receptor Alpha in Health and Disease
Source: Int J Mol Sci. 2023 Feb 21;24(5):4265. doi: 10.3390/ijms24054265 (PMC10002233; doi:10.3390/ijms24054265)
Supplement: Supplementary file 1 [file ijms-24-04265-s001.zip › ijms-2187977-supplementary.pdf]

Figure S1

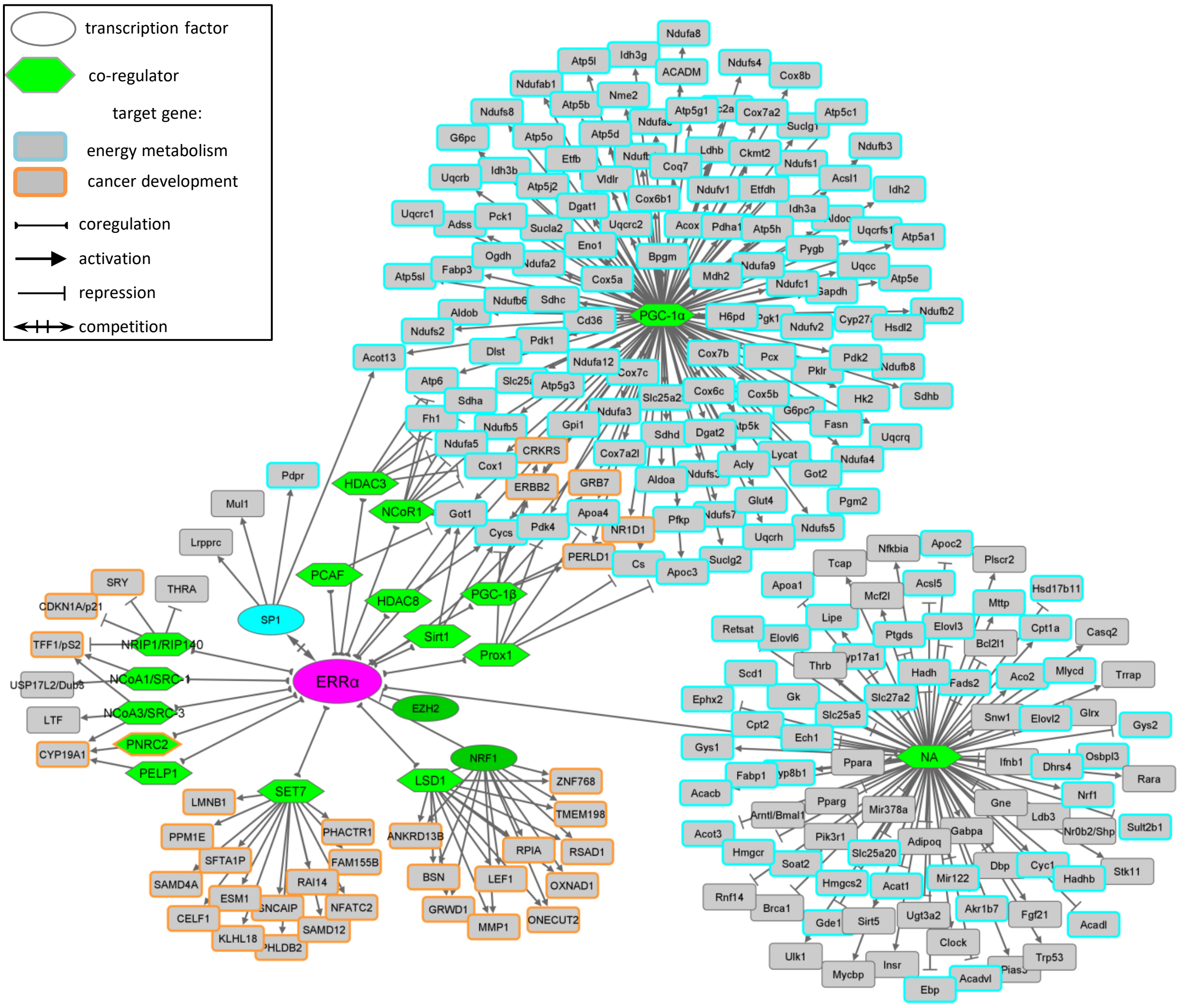

Figure S1: Transcriptional network of ERRα-coregulators complexes. ERRα and its coregulators, alone or in association, control the expression of specific genes involved in various biological functions, mostly energy metabolism or cancer development.

NA: unknown or unspecified coregulator.
